# Alkaline Phosphatase and Hypophosphatasia

**DOI:** 10.1007/s00223-015-0079-1

**Published:** 2015-11-21

**Authors:** José Luis Millán, Michael P. Whyte

**Affiliations:** Sanford Children’s Health Research Center, Sanford Burnham Prebys Medical Discovery Institute, La Jolla, CA 92037 USA; Center for Metabolic Bone Disease and Molecular Research, Shriners Hospital for Children, St. Louis, MO 63110 USA; Division of Bone and Mineral Diseases, Washington University School of Medicine at Barnes-Jewish Hospital, St. Louis, MO 63110 USA

**Keywords:** Rickets, Osteomalacia, Enzyme replacement, Calcification, Seizures

## Abstract

Hypophosphatasia (HPP) results from *ALPL* mutations leading to deficient activity of the tissue-non-specific alkaline phosphatase isozyme (TNAP) and thereby extracellular accumulation of inorganic pyrophosphate (PP_i_), a natural substrate of TNAP and potent inhibitor of mineralization. Thus, HPP features rickets or osteomalacia and hypomineralization of teeth. Enzyme replacement using mineral-targeted TNAP from birth prevented severe HPP in TNAP-knockout mice and was then shown to rescue and substantially treat infants and young children with life-threatening HPP. Clinical trials are revealing aspects of HPP pathophysiology not yet fully understood, such as craniosynostosis and muscle weakness when HPP is severe. New treatment approaches are under development to improve patient care.

## Introduction

In 1923, Robert Robison, Ph.D. discovered a phosphatase abundant in the skeleton possibly to generate inorganic phosphate (P_i_) required to form bone mineral [1]. In 1932, he postulated that an additional but unknown factor regulates skeletal mineralization [2]. This factor would prove to be inorganic pyrophosphate (PP_i_), a potent inhibitor of mineralization and natural substrate for Robison’s enzyme [3]. The phosphatase, now called tissue-non-specific alkaline phosphatase (TNAP, or TNSALP), is encoded by the *ALPL* gene [4] expressed richly in bone, liver, and kidney, but also in the central nervous system, fibroblasts, and endothelial and other cell types. Hypomorphic *ALPL* mutation(s) cause hypophosphatasia (HPP) [4], a rare form of rickets or osteomalacia [5] featuring low serum alkaline phosphatase (ALP) activity and with an incidence for its most severe forms of 1:100,000 [6] and 1:300,000 [7] in the general population [6] of North America and Europe, respectively, but 1 per 2500 births in Canadian Mennonites [8]. The efficacy of enzyme replacement therapy (EzRT) using a mineral-targeted form of recombinant TNAP has been demonstrated for severe HPP [9]. Our review summarizes knowledge concerning TNAP, including revelations about its physiological function coming from investigation of HPP patients and mouse models and successes using EzRT.

## The Enzyme

**Fig. 1:**
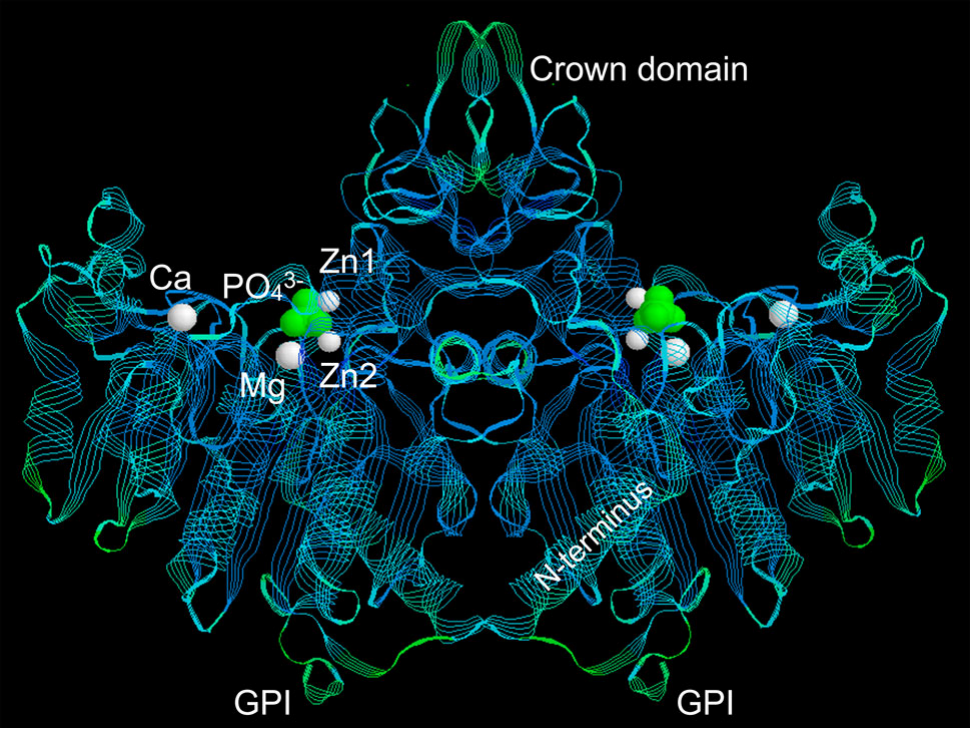
Ribbon representation of the 3D structure of alkaline phosphatase (1EW2) [10]. The active site phosphate (PO_4_^3−^) bound to Ser92 during catalysis is shown in *green*. The three active site metal ions, two Zn^2+^ (Zn1 and Zn2) and one Mg^2+^ (Mg), are shown in *white* as well as the structural Ca^2+^ ion (Ca). Also indicated are the flexible exposed sequence known as the “crown domain”; the N-terminal helix of one subunit that reaches close to the active site of the contralateral subunit; and the location where the glycosylphosphatidylinositol (GPI) anchor is attached to the C-terminus of the mature enzyme (Color figure online)

In humans, three genes besides *ALPL* (*ALPI*, *ALPP*, and *ALPPL2*) encode alkaline phosphatase (ALP) isozymes, but with restricted tissue expression; i.e., “intestinal,” “placental,” and “germ cell” ALP and are not compromised in HPP [10]. TNAP structure modeling is based on its sequence homology to the placental isozyme (PLAP) for which crystallographic coordinates have been determined [11]. TNAP functions physiologically as a homodimer (Fig. 1) [12]. The two monomers become related by a twofold crystallographic axis, with the monomer–monomer interface displaying a strong hydrophobic character with fewer than 30 % of its amino acid residues (hereafter “residues”) involved in hydrogen-bonding interactions [13]. This feature renders the monomer–monomer interface crucial for stability and enzymatic function, and TNAP (and mammalian ALPs in general) is therefore an obligatory homodimer. A flexible surface loop, “the crown domain,” contains residues important for stabilizing the binding of uncompetitive TNAP inhibitors, such as E429 and Y367 [14–16]. Furthermore, this domain contains a low-affinity collagen-binding motif [16]. The N-terminal α-helix (residues 9–25) of each monomeric subunit reaches the active site of the contralateral subunit (Fig. 1) [17]. Both the crown domain and the N-terminal arm help stabilize the dimeric structure and determine allosteric properties [18]. Thus, structural and functional properties explain how some hypomorphic *ALPL* alleles compromise the kinetic properties of the entire dimer (a dominant-negative effect) leading to TNAP insufficiency and generation-to-generation inheritance of HPP [19]. Three metal-binding sites surrounding the catalytic Ser residue are essential for TNAP enzymatic activity; i.e., M1 and M2 (occupied by Zn^2+^) and M3 (occupied by Mg^2+^) [11, 14]. An additional metal-binding site, M4, apparently occupied by Ca^2+^ and absent in bacterial ALPs, was revealed by solving the 3D structure of PLAP [11, 20]. However, this M4 structural metal site does not influence TNAP catalytic activity [21]. During TNAP-mediated catalysis, Zn^2+^ ions occupy the M1 and M2 sites, Mg^2+^ the M3 site, and Ca^2+^ the M4 site. With skeletal mineralization, the increasing Ca^2+^ gradient in the extracellular matrix first activates TNAP by replacing the Mg^2+^ with Ca^2+^ at M3, but at high Ca^2+^ concentrations, it gradually deactivates TNAP as Ca^2+^ competes out the Zn^2+^ from the M1 and M2 metal sites [21]. This explains why TNAP loses activity at the completion of the mineralization process [22].

Thus, *ALPL* mutations that alter residues at the monomer–monomer interface, the crown domain, the N-terminal arm, and the divalent cation-binding sites can all cause HPP [20]. Additionally, post-translational modifications of TNAP are important. TNAP (indeed all mammalian ALPs) is bound to the surface of plasma membranes via a glycosylphosphatidylinositol (GPI) anchor that enables movement of the enzyme by enhancing membrane fluidity [23]. This GPI anchor can be cleaved by the enzymatic action of phospholipases found in plasma membranes, perhaps explaining how TNAP is released into the circulation and other biological fluids [24]. Also, TNAP contains five putative N-linked glycosylation sites, N123, N213, N254, N286, and N413 [25], with the sugar chains required for catalytic activity [26]. The types of sugars in TNAP explain the different biophysical and kinetic properties of its “isoforms” produced by bone, liver, kidney, and vascular cells [27].

In vitro, TNAP has broad substrate specificity and can hydrolyze or transphosphorylate a considerable variety of compounds [10]. However, only a few [inorganic pyrophosphate (PP_i_), pyridoxal 5′-phosphate (PLP), and likely phosphoethanolamine (PEA)] are natural substrates for TNAP based on studies of HPP patients and fibroblasts and *Alpl* knockout (KO) mice [28, 29]. Nevertheless, it is unclear what metabolic pathway leads to PEA accumulation [27, 29]. Recent studies also suggest that ATP [30–32], di-phosphoryl lipopolysaccharide (LPS) [33], and phosphorylated osteopontin (p-OPN) [34] are natural substrates of TNAP.

## The Disease

**Fig. 2:**
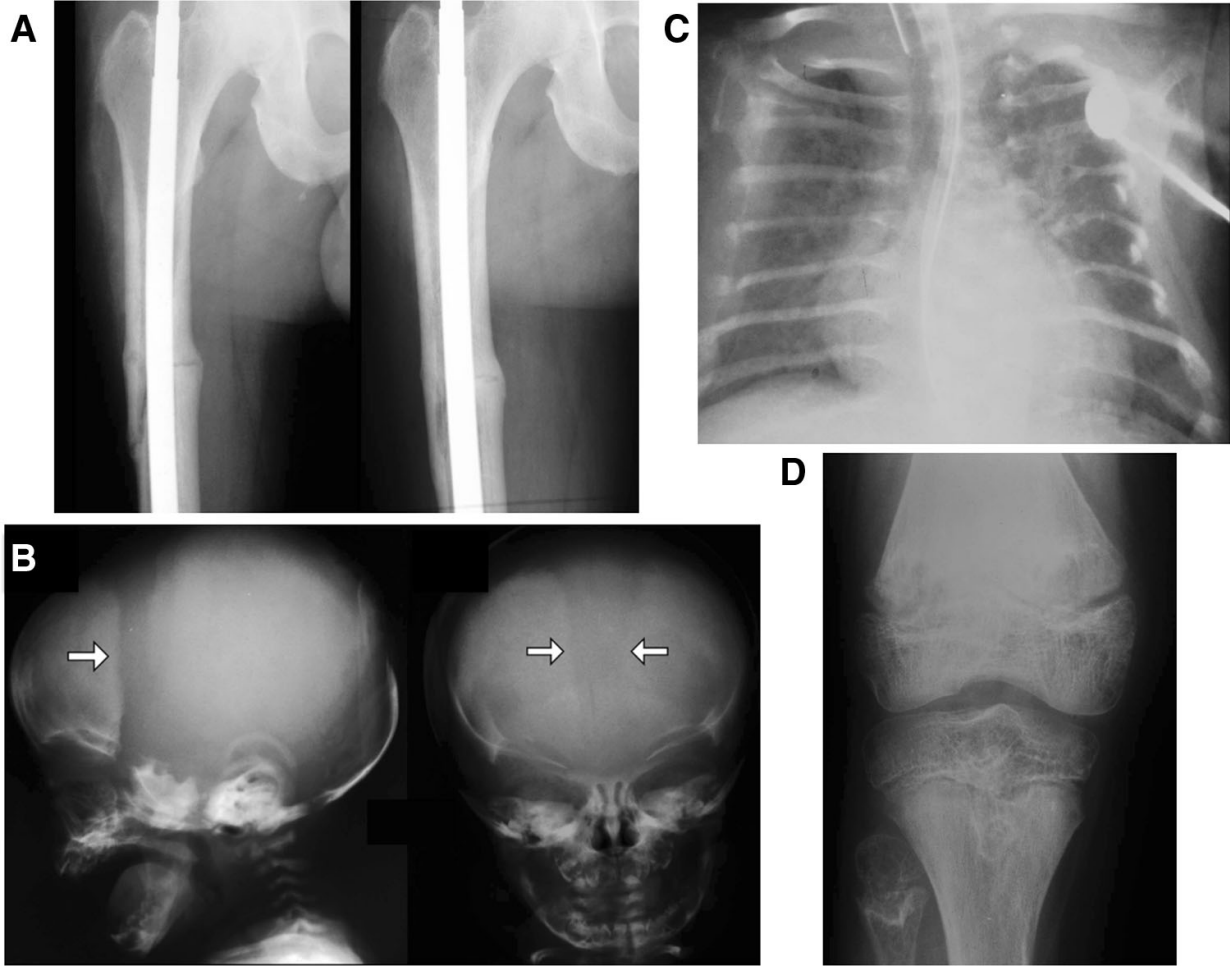
Radiographic images of HPP patients. **a** This middle-aged man who manifested signs and symptoms of hypophosphatasia during childhood shows some healing of a right femoral pseudofracture treated with intramedullary fixation. **b** Lateral and anteroposterior radiograph of the skull of this 14-week-old baby with infantile hypophosphatasia shows characteristic hypomineralization of areas of the calvarium that give the appearance of widened sutures (*arrows*). **c** At 23 weeks-of-age, this radiograph of the chest of a baby with infantile hypophosphatasia shows gracile, deformed, and fractured ribs that contribute to the respiratory compromise that is often lethal for such patients. **d** This radiograph of the right knee of a 9-year-old girl with childhood hypophosphatasia shows characteristic findings including areas of osteopenia and osteosclerosis especially in the metaphyseal regions, and marked physeal widening and irregularity of the head of the fibula

HPP features extraordinarily broad-ranging severity (Fig. 2), but all patients carry one or two loss-of-function mutations in their *ALPL* alleles [4, 35, 36]. Traditionally, HPP is classified in the clinic firstly by whether there are dental manifestations alone (i.e., without skeletal disease or other complications), and then according to the patient’s age when any additional complications initially manifested. Thus, in order of decreasing severity, clinicians recognize perinatal HPP, infantile HPP, childhood HPP, adult HPP, and odonto-HPP [35]. Perinatal HPP is detectable in utero using fetal sonography, and can cause stillbirth or be fatal soon after [37]. Infantile HPP presents before 6 months-of-age, often with rickets, failure-to-thrive, hypotonia, and muscle weakness, and can be complicated by hypercalcemia, nephrocalcinosis, vitamin B_6_-dependent seizures, and craniosynostosis [5, 38]. Vitamin B_6_-dependent seizures and skeletal deterioration predict death from respiratory complications [36]. If there is survival beyond infancy, deciduous teeth are lost prematurely (i.e., before the patient’s 5th birthday) because insufficiently mineralized cementum can not anchor their roots to the periodontal ligament [39]. Childhood HPP also has broad-ranging severity, but almost always includes premature loss of primary teeth. Rickets sometimes causes short stature, and skeletal deformities can include bowed legs and bony enlargement near joints due to widened metaphyses. Affected children often manifest some degree of motor impairment and fatigue easily. In 2015, “mild” versus “severe” childhood HPP were distinguished [36]. In occasional cases, childhood HPP presents in the guise of chronic recurrent multifocal osteomyelitis, possibly due to marrow edema secondary to pyrophosphate crystal deposition [40]. Adult HPP usually presents during middle age and features osteomalacia, although some patients report a history of rickets and/or early loss of “baby” teeth [41]. Recurrent, poorly healing, metatarsal stress fractures and then pseudofractures are typical skeletal complications. Some of these patients suffer calcium pyrophosphate dihydrate (CPPD) crystal deposition (chondrocalcinosis), PP_i_ arthropathy including attacks of pseudogout, or sometimes calcific periarthritis [42]. Odonto-HPP is diagnosed when the only clinical abnormality is dental disease, with radiological and biopsy studies revealing no evidence of rickets or osteomalacia. Figure 2 shows representative radiographic images of the skeleton in these principal forms of HPP.

HPP is a remarkable type of rickets or osteomalacia because circulating levels of calcium, P_i_, and vitamin D metabolites are not low. Instead, there is a block of mineral entry into the skeleton [35].

At this writing, 300 *ALPL* mutations have been identified in HPP, 70 % of which are missense (http://www.sesep.uvsq.fr/03_hypo_mutations.php). *ALPL* mutations can cause autosomal recessive (AR) or autosomal-dominant (AD) HPP. Perinatal and infantile HPP generally reflect compound heterozygosity for *ALPL* missense mutations [36], but sometimes there is homozygosity [9]. Childhood HPP reflects either AR or AD inheritance [35, 36]. Adult and odonto-HPP are usually caused by AD inheritance of *ALPL* alleles with dominant-negative effects [9, 19, 36].

## Mouse Models of HPP

In the 1990’s, two *Alpl* global KO mouse models for HPP (*Alpl*^*tm1Sor*^ and *Alpl*^*tm1Jlm*^), that differ in the design of the targeting construct, were developed independently by the research groups of Soriano [43] and of Millán [44]. In *Alpl*^*tm1Sor*^ KO mice, the genomic *Alpl* sequence spanning from the middle of exon 2 to the middle of exon 6 was replaced with a LacZ-Neo cassette to enable expression of β-galactosidase under control of the endogenous *Alpl* promoter. These mice had elevated plasma PLP levels and died from seizures caused by diminished hydrolysis of PLP to pyridoxal (PL) and thus deficient PL availability for cells of the central nervous system [45]. Surviving animals manifested dental dysplasia [43]. In contrast, the *Alpl*^*tm1Jlm*^ KO model was generated by inserting a Neo cassette into exon 6. Homozygous animals exhibited vitamin B_6_-dependent seizures and impaired bone mineralization [44]. Their lifespan averaged 8.8 ± 2.3 days on the 129J background, and 10.6 ± 3.4 days on the 129J, C57Bl/6J hybrid background. Plasma of *Alpl* KO mice contains little ALP activity, which comes from the gut [46], whereas heterozygous *Alpl*^*+/*−^ mice have approximately 50 % the plasma ALP activity of wild-type (WT) mice and are healthy and fertile [44].

**Fig. 3:**
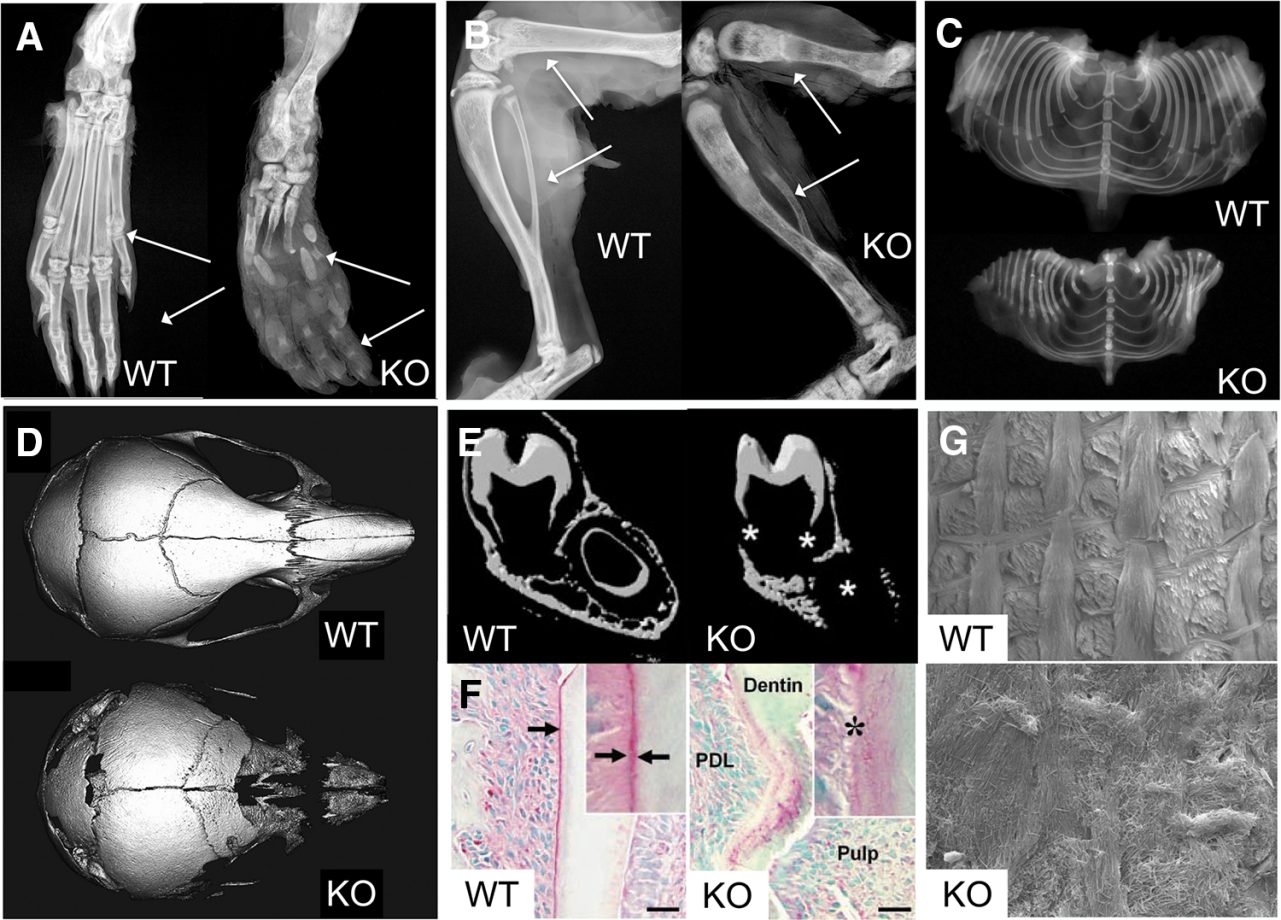
Phenotype of the *Alpl* ^*−/−*^ mouse model of infantile HPP. **a** X-ray images of a hind paw of a post-natal day 22 (P22) WT and *Alpl* ^*−/−*^ knockout (KO) mouse. **b** X-ray of the femur, tibia, and fibula of a P22 WT and KO mouse. Images from **a** and **b** were taken from [126] and reproduced with permission from BONE. **c** Radiographs of the ribs of a P16 WT and KO mouse. Images taken from [124] and reproduced with permission from the Journal of Bone and Mineral Research. **d** Micro-CT isosurface images of a P15 WT and KO mouse skull. Multiple cranial vault and facial bones are so severely hypomineralized in P15 that they do not appear on isosurface images calibrated to a bone threshold. The KO skull appears decreased in anterior–posterior length but increased in height when compared to the WT skull, and is more dome-shaped in overall appearance. Images taken from [100] and reproduced with permission from Bone. **e** Micro-CT demonstrates poorly mineralized molar roots and incisor in 10-day-old KO mice compared with WT, in addition to generalized reduction of bone mineralization in the mandible. Images taken from [47] and reproduced with permission from the Journal of Bone and Mineral Research. **f** Immunohistochemical localization of osteopontin (*red*, *arrows* and *inset*), as a marker for acellular cementum, shows a distinct line of acellular cementum in the WT sample, but an absence of a discrete immunostained layer in a 16-day-old KO mouse. *PDL* periodontal ligament, *En-S* enamel space after decalcification. Magnification bars equal 100 μm. Taken from [46] and reproduced with permission from the Journal of Dental Research. **g** Scanning electron microscopy (SEM) analysis of incisors (*top*) and molars (*bottom*) of WT and KO mice at 20 days-of-age. The SEM images show well-decussated enamel rods and inter-rod in the molar crowns and crown analogs of incisors of WT mice. Note that there is a lack of rod–inter-rod organization in the KO mice. Images taken from [48] and reproduced with permission from the Journal of Bone and Mineral Research (Color figure online)

In 1999, these two KO mouse models, *Alpl*^*tm1Sor*^ and *Alpl*^*tm1Jlm*^, were further characterized and compared [28]. Radiographic bone abnormalities appeared at ~10 days-of-age, and osteopenia and fractures worsened thereafter. Both had elevated urinary PEA and PP_i_ and plasma PLP levels. That same year, Beertsen et al. [47] reported a 2–3 day delay in incisors eruption and onset of mineralization of the mantle dentin within the developing molars of the *Alpl*^*tm1Sor*^ KO mice. In contrast, the dentin and enamel developed normally, except for some localized hypoplasia. The most conspicuous finding, however, involved the acellular cementum along the molar roots, which deposited as thin and irregularly shaped patches rather than a continuous layer around the base of the periodontal ligament fibers [47]. The *Alpl*^*tm1Jlm*^ KO mice had impaired dentin mineralization in the incisor and molar roots, ranging from a mild delay to severely disturbed dentinogenesis. Subsequently noted were lack of acellular cementum [48, 49] and disrupted organization of the rods and inter-rod structures in the enamel [50]. Also, premature fusion of the coronal suture (craniosynostosis) appeared in these *Alpl*^*tm1Jlm*^ KO mice [51]. Figure 3 shows representative radiographic images of their skeletal and dental phenotype. *Alpl*^*tm1Jlm*^ KO mice also have an apoptotic thymus, thin descending nerve roots, leukopenia, and gas accumulation in their small intestine [44, 52]. Most studies concerning murine HPP and the preclinical work for EzRT for HPP used *Alpl*^*tm1Jlm*^ mice. Accordingly, later, we simply refer to them as the KO mice.

Now, other *Alpl* mutant mouse lines have been generated, although their skeletal and dental phenotypes remain to be determined. In 2009, the *Alpl*^*ALPLD1*^ model was produced by ENU mutagenesis and carries an A to G mutation at nucleotide (nt) position 326 in exon 5 [53]. This causes an Asp to Gly change at residue 109. In 2012, Sabrautzki et al. reported new mouse models for various metabolic bone diseases produced by the Munich ENU Mutagenesis Project [54], including phenotypes associated with lowered plasma ALP activity due to mutations in *Alpl*. *Alpl*^*BAP023*^ carries a missense T to G mutaton in exon 7 at nt 755 (Leu to Pro at residue 251). *Alpl*^*BAP026*^ has a splice-site mutation in intron 9. *Alpl*^*BAP027*^ carries a T to A mutation in exon 10 at nt 1194 (Ile to Asn at residue 395). *Alpl*^*BAP032*^ has an A to G mutation in exon 11 at nt 1217 (Asp to Gly at residue 406). The homologous mutation had been found in an HPP patient. *Alpl*^*SAP007*^ has an A to G point mutation in exon 12 at nt 1357 (Thr to Ala at residue 453).

Currently, mouse models of adult HPP are receiving attention. In 2007, the Gena 328 mouse, *Alpl*^*Hpp*^, from ENU mutagenesis exhibited autosomal semi-dominant adult HPP [55]. A point mutation at the splice site for exon 8 produced a truncated, inactive TNAP having 276 residues rather than the 525 WT residues. However, some correct splicing was detected, suggesting the mutation is hypomorphic. These mice have low plasma ALP activity and late-onset skeletal abnormalities, but a normal life span and no epilepsy.

The first murine model of odonto-HPP harbors a missense mutation (c.346G>A) that changes codon 116 from Ala to Thr as identified in a kindred with AD odonto-HPP [56]. These *Alpl*^*+/A116T*^ mice have ~50 % WT plasma ALP activity and no differences versus WT mice in litter size, survival, or body weight. Their postcranial skeleton is normal radiographically, including no differences in femur length, cortical or trabecular structure or mineral density, or mechanical properties. However, their alveolar bone has radiolucencies and resorptive lesions and osteoid accumulation on the crest. Non-significant changes in acellular cementum seem not to affect periodontal attachment, although circulating ALP activity correlates significantly with incisor cementum thickness.

Now, two murine models of adult HPP reflect conditional ablation of *Alpl* in chondrocytes and osteoblasts together (*Prx1*-*Cre*; *Alpl*^*flox/flox*^), or in osteoblasts alone (*Col1a1*-*Cre*; *Alpl*^*flox/flox*^) (manuscript in preparation). Their phenotype compared to KO and *Alpl*^+*/A116T*^ mice is summarized in Table 1.

**Table 1:** Murine models of HPP spanning the severity of the disease

Manifestations	*Alpl* ^*−/−*^	*Alpl* ^*Prx1/Prx1*^	*Alpl* ^*Col1/Col1*^	*Alpl* ^+*/A116T*^
Seizures	Yes	No	No	No
Perinatal death	Yes	No	No	No
Rickets or osteomalacia	Yes	Yes	Yes	No
Joint defects	Yes	Yes	No	No
Alveolar bone hypomineralized	Yes	Yes	Yes	Yes
Absence of acellular cementum	Yes	Yes	Yes	No
Cellular cementum hypomineralized	Yes	Yes	Yes	Yes
Dentin (molar) hypomineralized	Yes	No	Yes	Yes
Dentin (incisor) hypomineralized	Yes	Yes	Yes	Yes
Enamel defects	Yes	?	?	Yes
Craniosynostosis	Yes	No	No	No
Nephrocalcinosis	Yes	No	No	No

?: not yet studied

## Pathophysiology of HPP

**Fig. 4:**
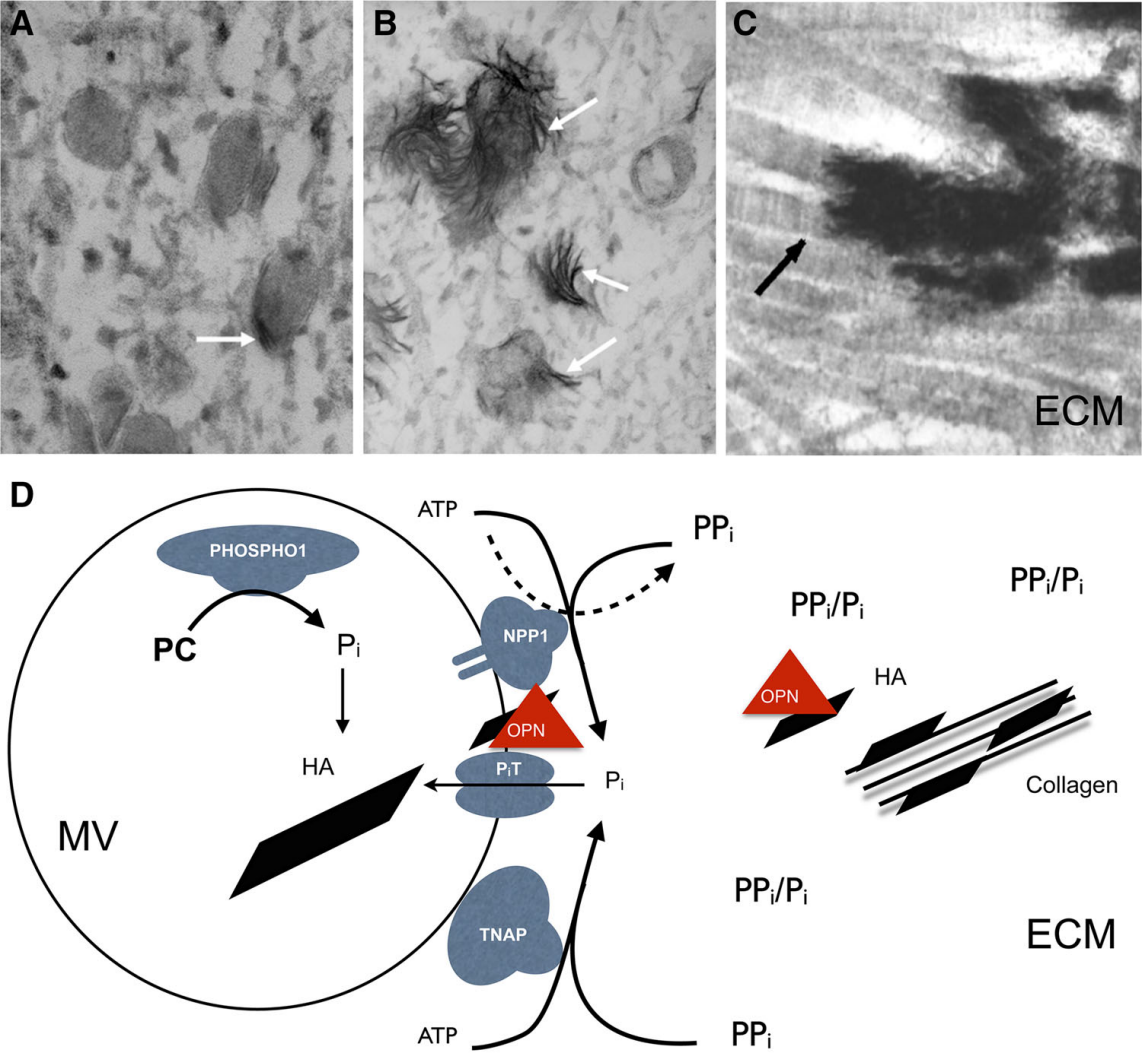
Mechanisms of initiation and propagation of skeletal mineralization. Scanning electron microscopy images of matrix vesicles (MV) displaying mineral confined to the interior of the vesicles (**a**), with mineral breaking through the MV membranes (**b**) and with mineral propagating onto the collagenous scaffold (**c**). **d** Diagram detailing our current understanding of the biochemical bases for these three steps of MV-mediated initiation of biomineralization. MVs appear to initiate HA mineral deposition by accumulation of P_i_ generated intravesicularly by the action of PHOSPHO1 on phosphocholine and also via P_i_T-1-mediated incorporation of P_i_ generated extravesicularly by TNAP or NPP1. The extravesicular propagation of mineral onto the collagenous matrix is mainly controlled by the pyrophosphatase activity of TNAP that restricts the concentration of this potent mineralization inhibitor and establishes a PP_i_/P_i_ ratio conducive for controlled calcification. Additionally, osteopontin (OPN), another potent mineralization inhibitor that binds to HA mineral as soon as it is exposed to the extracellular fluid, also controls the degree of extracellular matrix mineralization. Both elevated levels of PP_i_ and phosphorylated OPN are found in HPP mice. *ECM* extracellular matrix, *HA* hydroxyapatite, *OPN* osteopontin

In the skeleton, TNAP is confined to the surface of osteoblasts and chondrocytes, including their shed matrix vesicles (MVs) [57, 58] where the enzyme is particularly enriched [59]. In humans and mice with HPP (vide infra), electron microscopy revealed that TNAP-deficient MVs contain hydroxyapatite (HA) crystals, but extravesicular growth of these crystals is blocked (Fig. 4) by the extracellular accumulation of PP_i_ [3, 60–62]. Elevated urinary and plasma levels of PP_i_ were discovered in the 1960s [63–65]. Indeed, breeding *Alpl* KO mice to mice deficient in either the extracellular production of PP_i_ (*Enpp1*^*−/−*^) or transport extracellularly (*ank/ank*) of PP_i_ normalized extracellular levels of PP_i_. This prevented skeletal disease in the double KO mice, and affirmed PP_i_ accumulation as the cause of the rickets/osteomalacia in HPP [66, 67]. The effect of the double ablation was partial at some skeletal sites, likely reflecting variations of *Enpp1* expression [62, 66].

In the KO mice, ATP-dependent ^45^Ca precipitation was reduced within calvarial osteoblast-derived MVs and attributed to increases within the MV of PP_i_ from impaired hydrolysis [68]. However, TNAP is not only a PP_i_ase but, on MVs, is also a potent ATPase [31, 69]. Indeed, Yadav et al. showed that combined ablation of TNAP and PHOSPHO1 is additive and leads to the lack of HA crystal formation within MVs, the absence of skeletal mineralization, and embryonic lethality. PHOSPHO1 generates P_i_ inside MVs thus contributing to the initiation of HA mineral formation in the intravesicular space [70]. TNAP is a PP_i_ase crucial for allowing extravesicular growth of HA crystals, while also generating P_i_ from the hydrolysis of PP_i_ and acting as an extracellular ATP phosphohydrolase that generates P_i_ in the perivesicular space. Thus, current understanding of TNAP function supports Robison’s hypothesis concerning skeletal mineralization [35, 70, 71]. NPP1, the enzyme that when on the cell surface produces PP_i_, is also a potent ATPase in the MV microenvironment, and may act as a phosphatase in the absence of TNAP [30, 69–71]. Thus, NPP1 could be a modifier of the HPP phenotype, at least in mice. In fact, PHOSPHO1-deficient mice have increased plasma PP_i_ levels caused by reciprocal reduction of TNAP activity and increased NPP1 expression, as well as accumulation in the circulation of phosphorylated OPN, an inhibitor of mineralization that perhaps exacerbates the rickets/osteomalacia and fracturing of HPP. Because PHOSPHO1 modifies the HPP phenotype in KO mice, we have searched for *Phospho1* gene mutations in patients with HPP-like disease without *ALPL* mutations, however, thus far with negative results. Accordingly, the hypotheses proposed by Robison for skeletal mineralization [1, 2]: (i) TNAP as a P_i_-generating enzyme, and (ii) an unknown factor controlling the supersaturated milieu for mineralization (now recognized to be PP_i_), are validated by current data stemming largely from investigations of HPP [35, 72–75]. Furthermore, we currently appreciate that there are interactions between PHOSPHO1, NPP1, and TNAP during MV-mediated calcification: the first step is a convergence of two independent biochemical pathways: intravesicular P_i_ generation by the enzymatic action of PHOSPHO1, and the influx via P_i_ transporters of P_i_ generated in the perivesicular space by the enzymatic actions of TNAP and NPP1 (Fig. 4).

Another natural substrate of TNAP discovered by studying HPP patients is PLP, the major circulating form of vitamin B_6_. Vitamin B_6_, mediated by its various vitamers, is a cofactor for at least 110 enzymes. The three vitameric forms, pyridoxal (PL), pyridoxamine (PM), and pyridoxine (PN), can all be phosphorylated by PL kinase to their 5′-derivatives, PLP, PMP, and PNP, respectively [76, 77]. PLP is a coenzyme for the catabolizing of various amino acids, and for decarboxylations necessary for neurotransmitter generation including dopamine, serotonin, histamine, taurine, and gamma-aminobutyric acid (GABA). PLP and PMP are interconvertible through aminotransferases or PMP/PNP oxidase. Removal of P_i_ from PLP to form PL is one important function of TNAP [78]. Only the non-phosphorylated vitamers can enter cells, and once inside the cells, these non-phosphorylated vitamers are converted back to PLP to be used as a coenzyme for various enzymatic pathways. TNAP deficiency increases plasma PLP levels in all HPP patients, but leads to low levels of PL in the circulation in severely affected infants who consequently have insufficient incorporation of PL into the CNS and therefore vitamin B6-dependent seizures [45, 78–80]. Resembling the 100 % lethality when vitamin B_6_-dependent seizures occur in infantile HPP [38], KO mice too have 100 % mortality after weaning, always heralded by seizures. TNAP is highly expressed in the developing murine neural tube [81] and certain areas of the mature brain [82]. The absence of TNAP in KO mice is associated with hypomyelination and thinning of spinal nerves, the absence of myelinated axons, and an increased proportion of immature cortical synapses [44, 52, 83]. Matching clinical experience treating vitamin B_6_-dependent seizures in HPP, injection or ingestion of PL, a hydrophobic form of vitamin B_6_ that traverses biological membranes temporarily suppresses the epilepsy of KO mice [43, 44, 52].

PEA is elevated in the blood and urine of patients with HPP [28, 29, 45], but its endogenous origin is uncertain. PEA is a component of the cell-surface glycosylphosphatidylinositol link for proteins, including TNAP. However, an additional or alternative source of PEA may reflect diminished hepatic *O*-phosphorylethanolamine phospholyase (PEA-P-lyase) activity—the enzyme reported to hydrolyze PEA using PLP as a cofactor [84–86]. Because TNAP hydrolyzes extracellular PLP to PL to allow incorporation of PL into cells for the formation of PLP needed as a coenzyme, insufficient PLP inside hepatic cells could increase PEA accumulation. In this regard, the authors began their nearly career-long collaborations in 1979 when they studied a large multigenerational kindred with adult HPP who showed elevated PEA and phosphoserine levels in the urine which correlated inversely with serum total and liver TNAP activity, but not serum bone TNAP activity [87].

TNAP KO mice have high plasma osteopontin (OPN) levels encoded by *Spp1*, and elevated expression of its RNA in cultured osteoblasts [88–90]. Although the function of OPN is incompletely understood, it anchors osteoclasts to HA by its poly-aspartate sequences, while also binding CD44 and α_v_β_3_ integrin via its RGD sequence, thus mediating cell signaling and/or migration [91]. OPN is a highly phosphorylated glycoprotein, with 36 serine/threonine phosphorylation sites [92]. This phosphorylation is important because OPN’s inhibitory effect on mineral deposition diminishes if 84 % of this covalently bound phosphate is removed [93]. Phosphorylated, but not dephosphorylated, OPN inhibits mineralization in vascular smooth muscle cells [94]. Certain phosphorylated OPN peptides also inhibit HA formation in vitro [95], and cause dose-dependent inhibition of mineralization by cultured cells [96]. Extracellular PP_i_ levels control OPN expression by cultured osteoblasts [91, 96], and high plasma levels of phosphorylated OPN accompany the increased extracellular PP_i_ levels in *Alpl* KO mice [90]. In turn, [*Alpl*^*−/−*^; *Spp1*^*−/−*^] double KO mice have partial improvement of the hypomineralization compared to *Alpl*^*−/−*^ KO mice [90]. Hence, phosphorylated OPN accumulation may contribute to the impaired bone mineralization of *Alpl* KO mice. Absent TNAP function leads to accumulation of phosphorylated OPN [34]. This suggests that OPN is another substrate of TNAP, and OPN phosphorylation is an additional biochemical pathway in the pathophysiology of murine HPP (Fig. 4). We are currently reviewing the OPN status of HPP patients.

Interestingly, PHOSPHO1-deficient mice also have increased plasma levels of PP_i_ and phosphorylated OPN. Here, concurrent ablation of the OPN gene corrects the skeletal disease of *Phospho1* deficiency [97]. Thus, *Alpl* and *Phospho1* deficiency engender similar skeletal phenotypes and comparable changes in PP_i_ and OPN expression levels. However, there is a clear dissociation in the hierarchical roles of these potent inhibitors of mineralization, with elevated PP_i_ and phosphorylated OPN levels causing the respective skeletal phenotypes in *Alpl*^*−/−*^ and *Phospho1*^*−/−*^ mice.

**Fig. 5:**
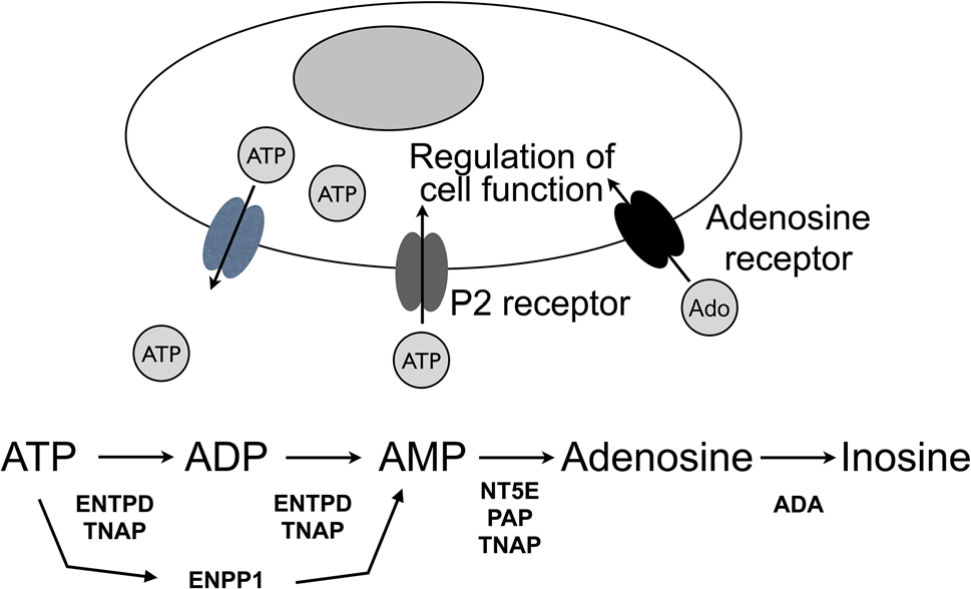
Enzymes involved in the catabolism of ATP to form adenosine, and thus regulate the ATP/adenosine ratio important for purinergic signaling. *ENPP1* ectonucleotide pyrophosphatase/phosphodiesterase, *ENTPD/CD39* ectonucleotide triphosphate diphosphohydrolase, *NT5E/CD73* ecto-5′-nucleotidase, *PAP* prostatic acid phosphatase, *ADA* adenosine deaminase, *TNAP* tissue-non-specific alkaline phosphatase

Other biological roles for TNAP are now being explored inspired by studies concerning intestinal ALP in protection of the gut mucosa and regulation of the gut microbiota via detoxification of bacterial endotoxins, especially di-phosphoryl-lipopolysaccharide (LPS) and pro-inflammatory ATP [98–100]. Lei et al. [33] showed in hamsters that LPS detoxification by TNAP conditions uterine receptivity for implantation and decidualization, while protecting the uterus and pregnancy against bacterial infection. High circulating TNAP activity in neonates enhances their high plasma levels of anti-inflammatory adenosine, produced by the dephosphorylation of pro-inflammatory ATP [31]. Furthermore, TNAP is one of three enzymes involved in purine metabolism, contributing anti-nociceptive adenosine for murine somatosensory dorsal root ganglia neurons and the dorsal spinal cord [32]. Altered purinergic signaling in *Alpl* KO mice could result from an increased ATP/adenosine ratio caused by TNAP deficiency. Such changes may contribute to the seizures, hyperalgia, and allodynia seen in *Alpl* KO mice, and perhaps patients with severe HPP (Fig. 5).

## What We Don’t Yet Understand About HPP

Premature fusion of cranial sutures (craniosynostosis) in HPP and other forms of rickets may seem counter-intuitive and remain unexplained. Patients with severe HPP receiving asfotase alfa still often require craniotomies [101] to relieve intracranial pressure. However, asfotase alfa [102] and ChimAP [103] (a soluble chimeric form of human ALP), both can prevent craniosynostosis, at least in mice (vide infra), if treatment starts at birth. So, prevention of craniosynostosis may depend on the time of initiation of the treatment, although there are data to indicate that in humans craniosynostosis may begin during embryonic life [104]. Thus, clarifying the onset and mechanism of craniosynostosis may help design interventions at a developmental stage when enzyme replacement may not be feasible in patients.

While the role of TNAP in the skeleton seems well understood, little is known about its function in the kidney, despite reference to this ALP isozyme as liver/bone/kidney type ALP. Expression of TNAP in the kidney may seem counter-intuitive because TNAP hydrolyses PP_i_, a potent mineralization inhibitor that could help prevent kidney stones. In fact, reduced urinary PP_i_ predisposes to nephrolithiasis [105]. PP_i_:creatinine ratios were reduced in 107 patients with recurrent calcium kidney stones [106]. PP_i_ levels in urine are >10 µM compared to plasma concentrations of approximately 1-6 µM that mainly arise from liver metabolism [107]. Intravenous ^32^PP_i_ is rapidly hydrolyzed in plasma, with PP_i_ also being filtered at the glomerulus and subject to further hydrolysis within the kidney; <5 % of intravenous ^32^PP_i_ appears in urine. These data indicate that PP_i_ in the kidney likely originates locally. Thus, nephrocalcinosis despite high urinary concentrations of PP_i_ in severely affected babies with HPP could seem puzzling, but their severe hypercalcemia and hypercalciuria is likely the key factor. Interestingly, children and adults with HPP are often hyperphosphatemic with increased TmP/GFR, suggesting kidney TNAP plays a role in P_i_ excretion [36].

## Supportive Management of HPP

HPP management has been, until very recently, supportive [9]. Bone-targeted enzyme replacement therapy (asfotase alfa, now Strensiq™) was approved for HPP in Japan (July, 2015), and for pediatric-onset HPP in Canada (August, 2015), Europe (August, 2015), and the USA (October, 2015). Supportive treatment includes hydration and restriction of dietary calcium and perhaps administration of calciuretics for infants with hypercalcemia and hypercalciuria [35, 36]. Management of the respiratory complications of perinatal and infantile HPP may be complex as multiple factors compromise their pulmonary function [108]. Vitamin B_6_-dependent seizures can respond temporarily to pyridoxine administration. Craniosynostosis may need surgical intervention. Fractures can mend, but often slowly and requiring prolonged casting or stabilization with intramedullary hardware. Femoral pseudofractures can also respond to intramedullary rodding [109]. Dental manifestations can be helped by a knowledgeable dentist.

## Targeting TNAP to Bone Mineral

Bolstered by insight derived from discovery of elevated plasma PLP levels in HPP [45] that predicted TNAP is cell-surface anchored, intravenous administration of ALPs to HPP patients was attempted to decrease extracellular PP_i_ levels and thereby improve skeletal mineralization. However, experience with such EzRT, including plasma from patients with Paget bone disease containing high bone TNAP activity, proved unsuccessful [110]. A 6-month-old girl with worsening infantile HPP repeatedly given intravenous infusions of TNAP-rich plasma obtained by plasmapheresis from two men with Paget bone disease showed circulating ALP activity with a half-life of ~2 days that persisted during a 5-week period of six ALP infusions. Sequential radiographic studies were interpreted as showing arrest of worsening rickets with slight remineralization of metaphyses, although urinary levels of PEA and PP_i_ seemed unaltered [110]. However, three subsequent patients deteriorating from infantile HPP [111] and given similar infusions of Paget plasma showed no radiographic or other benefit. Discouraging findings also occurred with attempted EzRT using purified human liver TNAP [112] and purified PLAP [113]. The cumulative findings indicated necessity for increasing TNAP activity in situ in the skeleton to reverse the pathophysiology of HPP. This hypothesis was supported in 2003 and 2007 by rescue with radiographic improvement demonstrated by two unrelated girls with life-threatening infantile HPP following attempts to transplant healthy mesenchyme-derived marrow cells, hoping TNAP-replete donor osteoblasts or chondrocytes might engraft in their skeletons [114, 115]. Subsequently, a woman with adult HPP carrying the most common American *ALPL* mutation seemed to benefit from teriparatide injections given to increase TNAP biosynthesis by her osteoblasts [116]. Then, anabolic treatment with parathyroid hormone 1-34 or 1-84 benefitted some adults with HPP [117–119], but not others [120, 121]. Parathyroid hormone therapy is not sanctioned for children, because osteosarcoma appeared in growing rats given teriparatide [122]. Evaluation of anti-sclerostin antibody (BPS804) therapy, which has been reported to increase bone-specific ALP when administered to healthy post-menopausal women [123] (www.clinicaltrials.gov identifier NCT01406977), was not carried forward. Anabolic approaches may favor patients with heterozygous *ALPL* mutations, because the WT *ALPL* allele is intact for upregulation; however, it will probably not be useful for severe AR HPP with at best “more of a bad enzyme.”

**Fig. 6:**
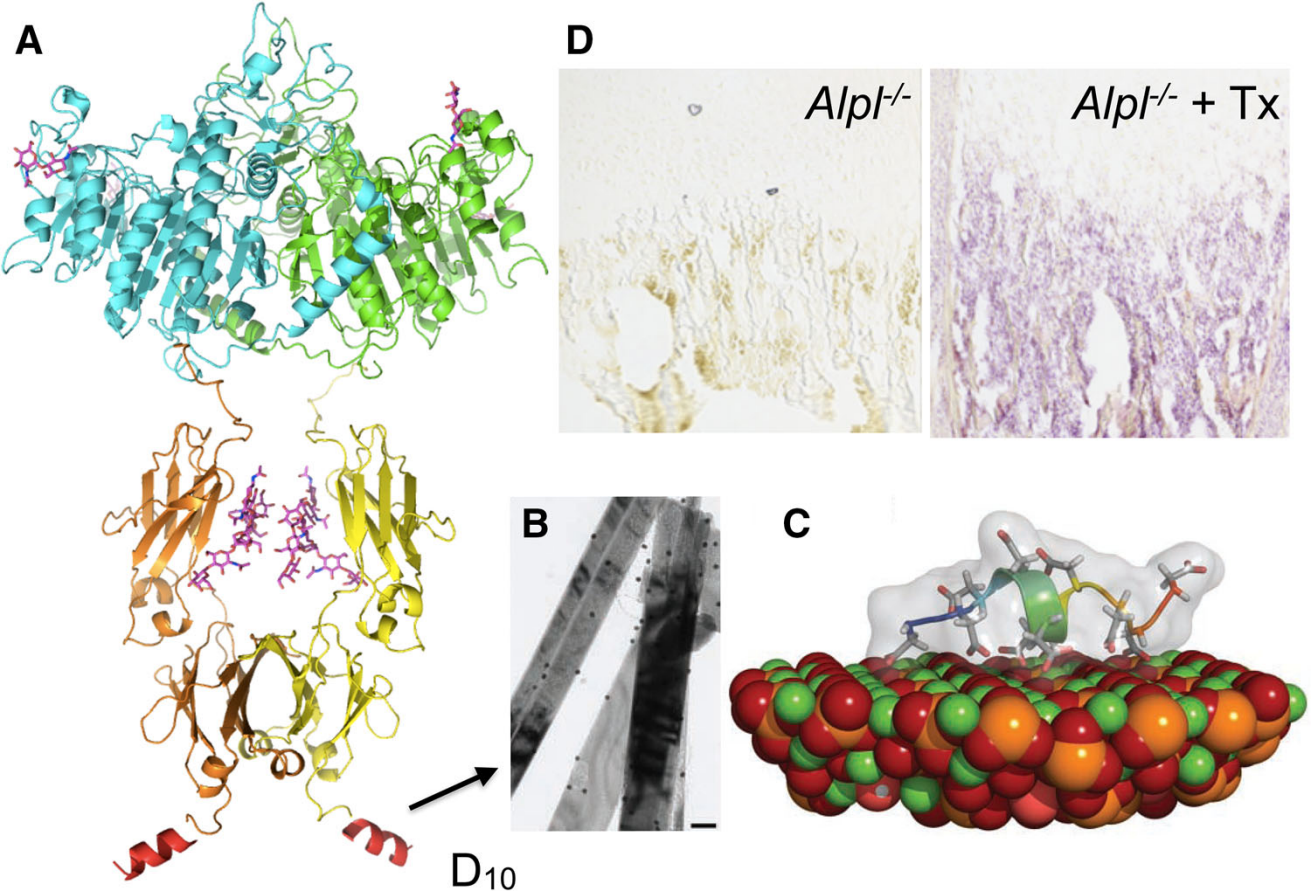
Structure and binding of asfotase alfa to bone mineral. **a** Three-dimensional modeling of ENB-0040. The model shows rigid ALP and Fc modules connected by a highly flexible linker. The terminal poly-Asp region is exposed on the opposite site of the ALP module. The whole structure is dimeric that conforms to the preferred oligomeric state of the ALP as well as the Fc region of the antibody. The three active site metal ions (two Zn^2+^ and one Mg^2+^) are marked with *blue spheres*. **b** Transmission electron micrograph showing the binding of sALP-FcD_10_ to synthetic hydroxyapatite crystals as revealed by immunogold labeling (*inset* is control incubation without sALP-FcD_10_ showing an absence of gold-particle labeling). Magnification bar equals 100 nm. **c** RosettaSurface-simulated model of D_10_ binding to a calcium-rich plane of the [100] crystallographic face of hydroxyapatite. **d** Histochemical staining for ALP activity in long bones of an ENB-0040–treated *Alpl* KO mouse compared with an age-matched untreated *Alpl* KO mouse. Images shown in **b** and **c** were taken from [46] (Color figure online)

In 2005, Enobia Pharma, Montreal, Canada bio-engineered and then expressed in Chinese hamster ovary (CHO) cells a first-in-class mineral-seeking recombinant TNAP to treat HPP. This biologic reflected in tandem the coding sequences for (i) soluble human TNAP, by excluding the hydrophobic C-terminal GPI anchoring motif (i.e., soluble TNAP: sALP), (ii) the Fc region of human IgG gamma-1 (Fc) to facilitate purification and prolong the circulating half-life, and (iii) ten acidic aspartate residues (D_10_) for selective drug delivery to bone [124–126]. The resulting 726-amino acid fusion protein was initially designated sALP-FcD_10_ to indicate its different domains (Fig. 6), renamed ENB-0040 when produced under Current Good Manufacturing Practice (cGMP), and asfotase alfa after clinical trials began. Alexion Pharmaceuticals, Cheshire, CT acquired Enobia Pharma in 2012. Asfotase alfa is now also called Strensiq™.

## Preclinical Evaluation of Bone-Targeted EzRT for HPP

Preclinical testing of sALP-FcD_10_ primarily involved *Alpl*^*tm1Jlm*^ (KO) mice. Pharmacokinetics (PK) and tissue distribution evaluations included newborn and adult mice and comparisons of different routes of administration [127]. Histochemical staining confirmed localization and catalytic activity of sALP-FcD_10_ in bone tissue (Fig. 6).

The first study involved newborn KO mice given subcutaneous (SC) injections for 15 days, then investigated with micro-computed tomography (µCT) of the calvarium and proximal tibial growth plate [127]. At 2 mg/kg daily, general appearance, body weight, and tail length improved with normal growth. At 8.2 mg/kg daily, body weight was greater than control mice, and plasma PP_i_ concentrations were normal. sALP-FcD_10_ minimized hypomineralization in the feet and reduced the number of mice with severely dysmorphic rib cages. The hind limbs appeared healthy in all treated animals [127]. A 52-day study of daily SC injections of 8.2 mg/kg also showed normal plasma PL and calcium concentrations and prevention of skeletal defects and epilepsy with good survival [127]. Hypomineralization of dentin and alveolar bone was also prevented and acellular cementum now formed properly [48, 49]. Immunohistochemical staining for OPN revealed unremarkable, rather than absent, acellular cementum on root surfaces [48]. The KO mouse dentin defect results from inability of MV-initiated mineralization foci to expand into a mineralization front, and varies from delayed mineralization to arrest of mantle dentin mineralization together with lack of circumpulpal dentin and odontoblast differentiation defects [49]. The OPN accumulation likely contributes to the impaired mineralization of mantle dentin. Early treatment with sALP-FcD_10_ enabled dentinogenesis and molar mineralization.

**Fig. 7:**
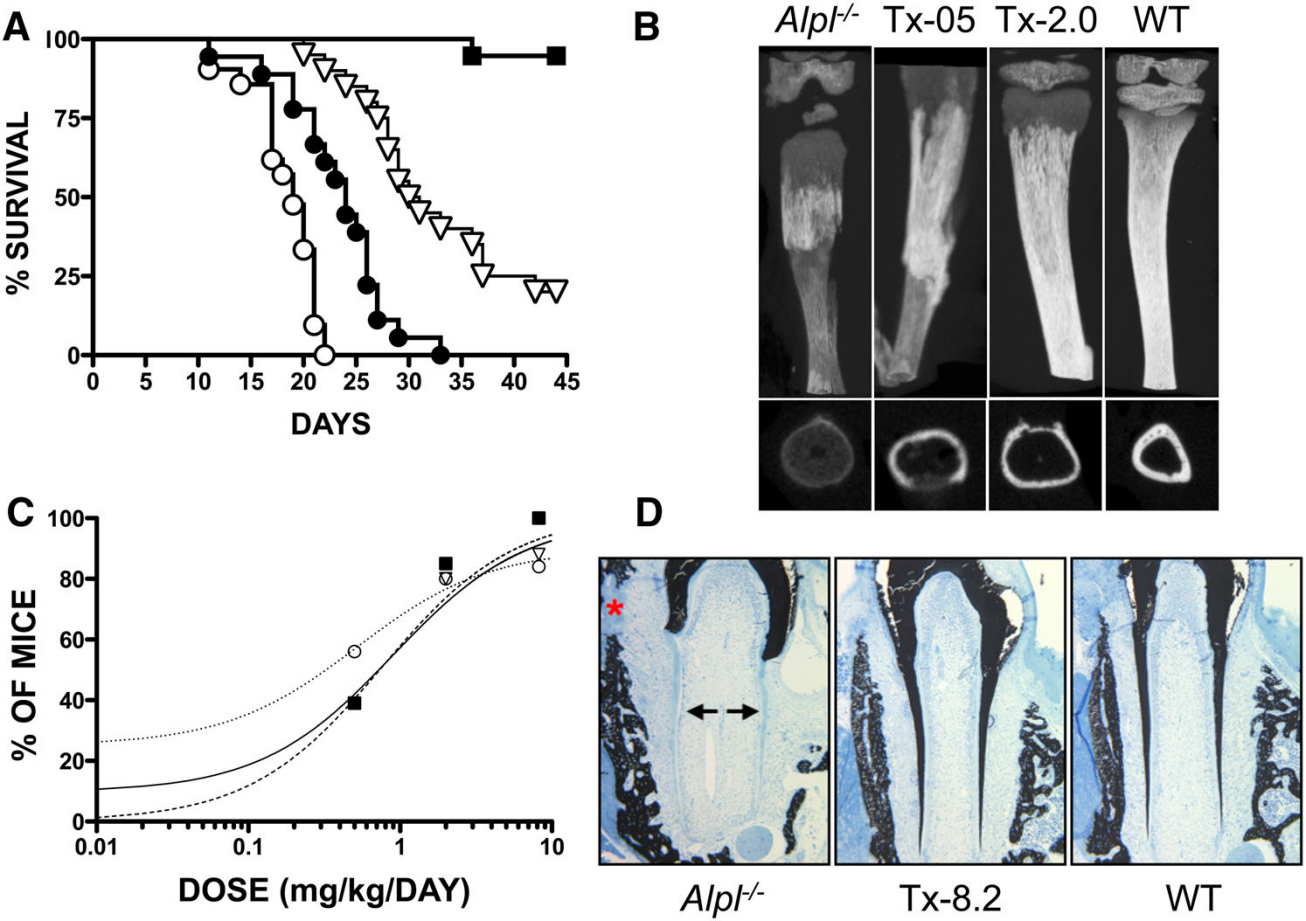
Preclinical efficacy of asfotase alfa. **a** Percentage survival of *Alpl* ^*−/−*^ mice receiving either vehicle (*white circle*) or escalating doses of asfotase alfa, i.e., Tx-0.5 (*black circle*), Tx-2.0 (*white down-pointing triangle*), or Tx-8.2 (*black square*). **b** μCT images of tibiae of the 22-day-old *Alpl* ^*−/−*^ mice treated with vehicle, Tx-0.5, Tx-2.0, and untreated WT mice. The images clearly show improved tissue mineral density and callus formation at the site of fractures in the treated mice. Transaxial views at the bottom. **c** Percentage of *Alpl* ^*−/−*^ mice considered normal as a function of asfotase alfa dose for feet (*black square*), rib cage (*white down-pointing triangle*), and lower limbs (*white circle*). **d** Asfotase alfa treatment maintains complete mineralization of all molar dentin as well as the surrounding alveolar bone such that no mineralization differences are seen between the molar teeth and bone of the treated *Alpl* ^*−/−*^ mice (8.2 mg/kg/day) compared with WT mice. Images taken from [128] with permission from BONE

Then, the relationship was defined between sALP-Fc-D_10_ doses and therapeutic responses after 43 days [128] (Fig. 7). Endpoint assessments included survival, body weight, tibial and femoral length, and bone mineralization in the feet, rib cage, and lower limbs assessed radiographically. Radiographs, µCT, and histomorphometry evaluated skeletal mineralization. Daily dose correlated clearly with the percentage of normal feet, rib cages, and lower limbs. An effective dose was established for 80 % of the mice (ED_80_) of ~3.2, ~2.8, and ~2.9 mg/kg/day for feet, rib cage, and lower limbs, respectively. The ED_80_, along with serum ALP activity and sALP-FcD_10_ PK data were used to estimate the minimum effective dose for these KO mice.

Further studies then validated the mineral targeting, including to sites notorious for poor vascularity, e.g., the enamel organ during tooth development. Untreated KO mice showed with scanning electron microscopy disorganization of the rod and inter-rod structures of enamel. Histology revealed enamel hypomineralization both in molars and incisors, loss of polarization of ameloblasts important for enamel matrix formation, and even absent enamel. What was now called ENB-0040 reached the enamel organ during its secretory and maturation phases [50]. µCT showed, with treatment to age 23 days, dose-dependent improvements of absolute and relative enamel volumes. Notably, odonto-HPP typically manifests the mildest reductions of serum ALP levels [35], usually explained by dominant inheritance of a single mutated *ALPL* allele [19], and reveals that tooth formation, especially cementogenesis, is the developmental process most sensitive to TNAP function, involving changes in the local P_i_/PP_i_ ratio [129, 130]. ENB-0040 treatment preserved acellular cementum formation in the KO mice, supporting this premise [48] and that the structural integrity of enamel by the enamel organ is directly regulated by extracellular PP_i_ concentrations [50]. TNAP is highly expressed in mature odontoblasts, and the molar and incisor roots of KO mice feature dentin hypomineralization ranging from mild to severe. Lack of mantle dentin mineralization accompanies disordered and dysmorphic odontoblasts. However, in KO mice, the formation of, initiation of mineralization within, and rupture of MVs in dentin matrix are not compromised. ENB-0040 corrected the defective dentin mineralization in the molar roots [49]. Liu et al. [51] showed that these mice by age 3 weeks have craniofacial shape abnormalities suggestive of limited anterior–posterior head growth with craniosynostosis (i.e., bony coronal suture fusion). μCT isosurface images showed ENB-0040 8.2 mg/kg/day from birth prevented at age 15 days craniofacial bone mineralization defects and premature suture fusion. In untreated KO mice, multiple cranial vault and facial bones lacked adequate mineralization on μCT that was not evident in the treated mice. Digital caliper linear measurements demonstrated that treatment improved nose length, nasal bone length, and frontal bone length [102].

## Clinical Trials Using Mineral-Targeting TNAP in Pediatric HPP

In June 2008, an Investigational New Drug (IND) application to test ENB-0040 for HPP patients was filed by Enobia Pharma. Initial dosing was based partly on (i) dose–response data in KO mice [128], (ii) efficacy observed in treated mice that achieved an equivalent to serum ALP activity in the range of 2400–6000 U/L; (iii) the No-Observed-Adverse-Effect Level (NOAEL) in more sensitive species (rats and monkeys) established in 1-month IV toxicology studies, and 1-month IV/SC bridging and tolerability studies in rats; and (iv) a safety factor of 10 applied to the NOAEL.

The first results in HPP patients were from an open-label, multicenter center, dose-escalating phase 1 study of the safety, tolerability, and pharmacology of ENB-0040 in six adults with HPP (www.clinicaltrials.gov identifier NCT00739505). They received one dose iv of 3 mg/kg, and then 1 or 2 mg/kg sc once weekly for 3 weeks.

**Fig. 8:**
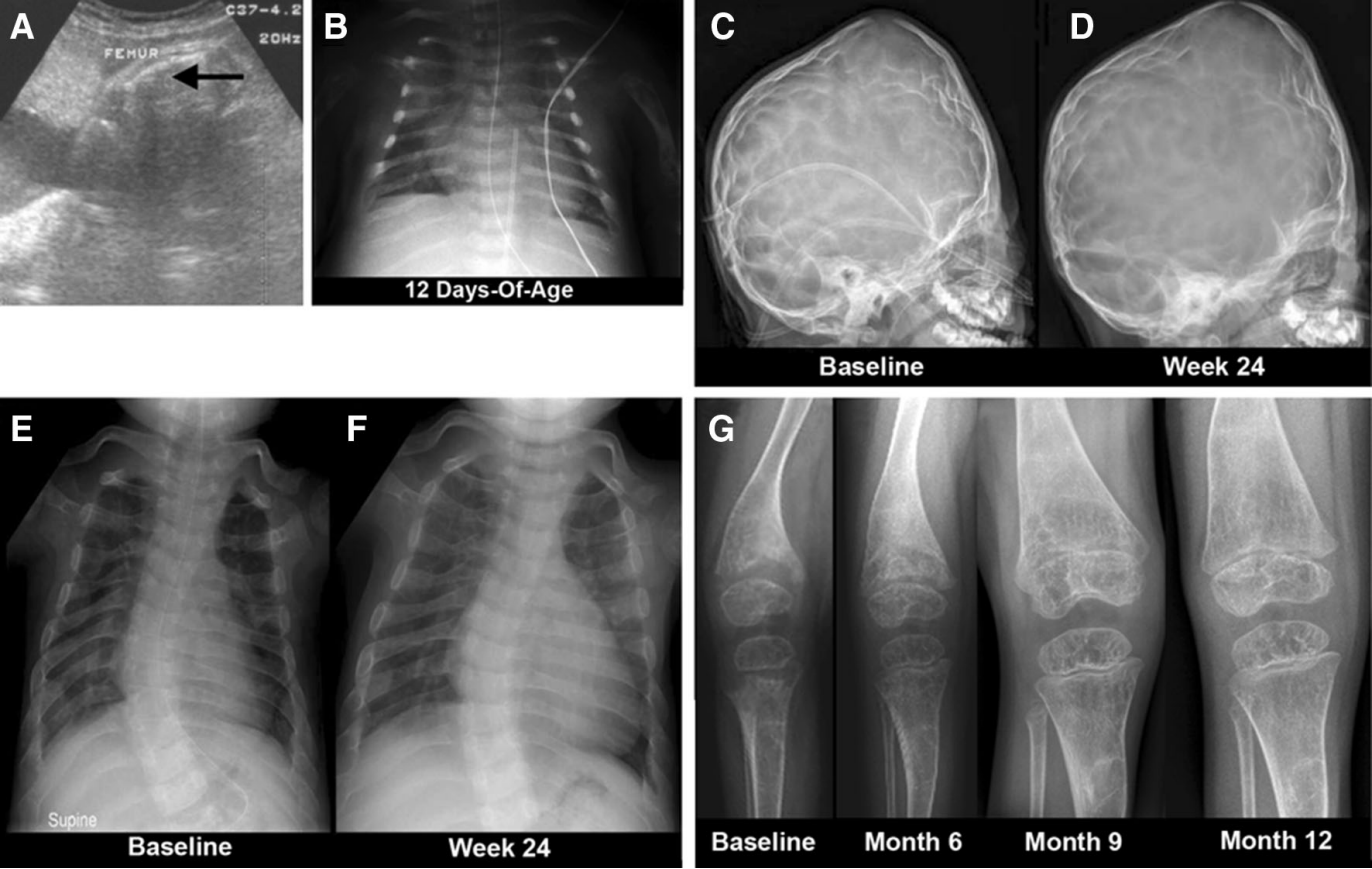
Efficacy of asfotase alfa treatment in a 36-month-old girl (at therapy baseline) with life-threatening HPP. **a** She has a short, bowed femur detected in utero by ultrasound. **b** At 12 days-of-age, her chest radiograph showed thin, osteopenic ribs with lytic areas and fractures. **c**, **d** Images of the skull before and after 24 weeks of ENB-0040 treatment. Note the severe pan-suture closure, including a marked increase in “digital” markings (“beaten-copper” appearance). **e** The ribs at baseline were osteopenic and had fracture deformities with thin cortices. **f** By week 24 of treatment, the ribs were wider and better mineralized with sharper cortical margins and less deformity. **g** Improvement of the rickets with therapy is apparent. Images taken for the online supplementary data in [9] and reproduced with permission from The New England Journal of Medicine

**Fig. 9:**
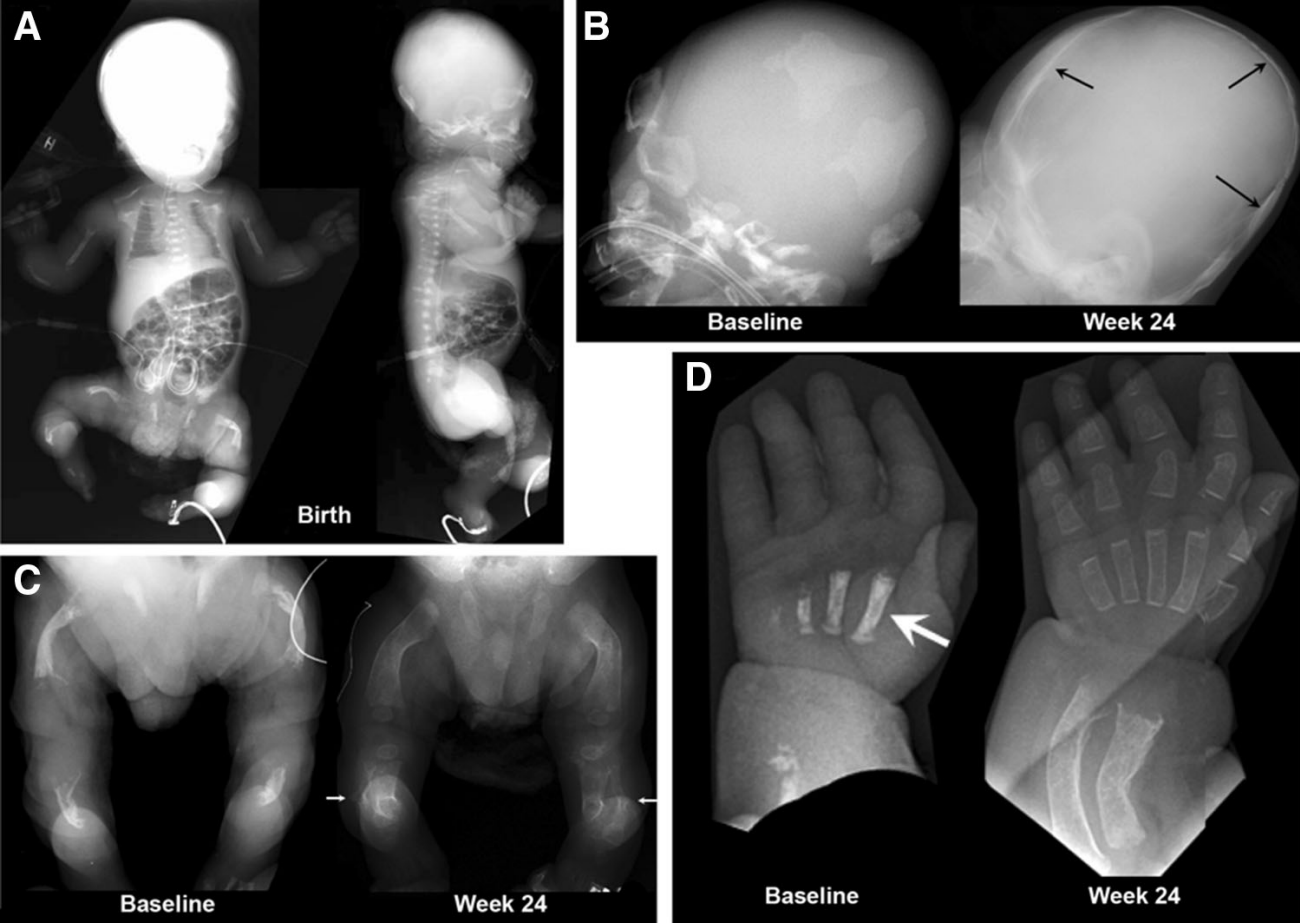
Efficacy of asfotase alfa in a 20-day-old (at therapy baseline) patient with life-threatening HPP. **a** This boy had shortened and bowed extremities and “fractures” detected by prenatal sonography at 17–18 weeks gestation. **b** Before asfotase alfa treatment, little or decreased mineral was present in the frontal, parietal, or occipital bones, skull base, facial bones, and sphenoid. **c** Before asfotase alfa, the femora were short, sclerotic, bowed, irregular, and lacked defined medullary cavities, cortices, and mineralized metaphyses and epiphyses. The fibulae were not calcified. After therapy, striking mineralization was evident. At week 24 of therapy, all areas showed striking remineralization. **d** The improvement in the left hand and wrist was remarkable. Images taken for the online supplementary data in [9] and reproduced with permission from The New England Journal of Medicine

The first full report concerning HPP treatment was from an open-label, multicenter, international study published in 2012 [9]. It evaluated the safety, tolerability, bioavailability, PK, pharmacodynamics, and efficacy of asfotase alfa given for nearly 1 year to infants and young children ≤3 years-of-age with life-threatening perinatal and infantile HPP. Before treatment, one 3-year-old girl had lost nearly all radiographically apparent skeletal mineral. The Supplementary Appendix to that paper provides a detailed description of each patient [9]. Efficacy assessments included changes in radiographic scales to evaluate the characteristic skeletal findings, gross and fine motor function, cognitive development, and pulmonary complications and their management. Asfotase alfa was administered as a single iv infusion of 2 mg/kg, followed by sc injections of 1 mg/kg thrice weekly for 24 weeks, with an extension study thereafter. The sc dose could be increased up to 3 mg/kg for worsening failure-to-thrive, deteriorating pulmonary function, or no radiographic evidence of skeletal improvement [9]. Of eleven recruited patients, parents withdrew one because she had a moderate adverse reaction during the iv infusion. One infant who completed the first 6 months of treatment died soon after from pneumonia and sepsis judged unrelated to the study drug. Of the nine patients treated for 1 year, four represented perinatal HPP and five represented infantile HPP. Typically, serum calcium levels were elevated (most had nephrocalcinosis), and dietary calcium had been restricted in all but one patient. During treatment, substantial radiographic improvement in skeletal abnormalities was documented at week 24 in all but one patient, with continued healing through week 48. The girl with the most extreme skeletal disease eventually improved with calcification apparent after 9 months of therapy and the delay probably reflecting her profound deficit of skeletal mineral. The radiographic improvement is illustrated for the oldest patient (3 years old) (Fig. 8), and for the youngest patient (<1 month old) (Fig. 9). Positive mineral balance throughout the skeleton was obvious radiographically, sometimes after several weeks or months, and involved diffusely membranous as well as endochondral bone. Symptomatic hypocalcemia from “hungry-bones” did not occur, but serum calcium decreased in some patients, consistent with improved uptake of calcium into mineralized bone. Increases in serum parathyroid hormone levels called for liberalization of dietary calcium intakes [9]. Deciduous teeth erupted in all the patients during therapy, with only one patient subsequently having HPP-related tooth loss [9]. Management of perinatal HPP in particular is critically dependent on early diagnosis [131]. An important concern is that some clinical laboratories do not flag as abnormal low serum ALP and this can delay the diagnosis and increase the risk of severe respiratory morbidity. Institution of EzRT should also not be delayed in order to correct any vitamin D deficiency—this can be accomplished contemporaneously if necessary.

Subsequently, asfotase alfa was given for 5 years to 12 children who were 5–12 years of age at study entry and substantially impaired by HPP [132]. Two radiographic scales to quantitate HPP skeletal disease documented rapid and significant improvement at 6-months of treatment, including comparisons to serial radiographs from similarly affected historical controls. Further improvements included patient growth, strength, motor function, and agility that achieved normal values for age- and gender-matched peers and were sustained at 5 years of therapy. Pain and disability resolved for most patients [132]. Mild-to-moderate injection site reactions were common, and sometimes associated with lipohypertrophy. Low titers of anti-asfotase alfa antibodies were noted in all. No evidence emerged for clinically significant ectopic mineralization or resistance to the treatment [132].

## Future Treatments for HPP

Kiffer-Moreira et al. recently evaluated ChimAP in KO mice. ChimAP is a chimeric ALP engineered by substituting the flexible crown domain of human IAP by that of human PLAP [133]. A clinical study is currently underway to assess ChimAP for treating acute kidney injury (www.clinicaltrials.gov identifier NCT02182440). Daily SC injections of ChimAP to KO mice at doses of 1, 8, or 16 mg/kg from birth to age 53 days were associated with normal lifespan and body weight and prevention of vitamin B6-dependent seizures at 16 mg/kg/day [103]. Radiographs, μCT, and histological analyses documented sustained mineralization of cortical and trabecular bone and secondary ossification centers in long bones. Craniosynostosis was prevented, and no evidence emerged of ectopic calcification by radiography and histology of the aorta, stomach, kidneys, or lungs [103]. AM Pharma (Bunnik, The Netherlands) has recently received FDA and EMA orphan drug designation for investigation of ChimAP (RecAP) for HPP. Daily sc injections of asfotase alfa are required to prevent HPP in mice, whereas nearly daily or thrice weekly sc injections are used to treat HPP patients. Viral vector delivery of mineral-targeting TNAP by this type of gene therapy might be an alternative to repetitive injections.

In 2011, Yamamoto et al. [134] demonstrated that a single sc iv injection of a lentiviral vector expressing mineral-targeting TNAP at birth permanently increased plasma TNAP levels in KO mice that survived more than 10 months and had normal physical activity, healthy appearance, no epileptic seizures, and radiographs showing significantly improved or preserved skeletal mineralization using this gene therapy. Also in 2011, Matsumoto et al. [135] demonstrated similar treatment effects, but using the adeno-associated virus serotype 8 (AAV8) vector that is more promising than lentiviral vectors for clinical trials. They expressed mineral-targeted TNAP and soluble, non-targeted TNAP tagged with the FLAG epitope. A single IV injection of 5 × 10^10^ vector genomes of either TNAP into KO mice at day 1 of life prolonged survival and prevented the skeletal abnormalities, suggesting that sustained ALP activity in the circulation at some threshold level may prevent the manifestations of HPP.

In 2012, Sugano et al. [136] demonstrated the feasibility of fetal gene therapy for HPP by giving KO mouse fetuses, at 15 days gestation, a single transuterine intraperitoneal injection of AAV serotype 9 (AAV9) expressing mineral-targeted TNAP. After birth, these mice showed normal weight gain and seizure-free survival for at least 8 weeks. ALP activities in plasma and bone were consistently high, and sustained mineralization was demonstrated on radiographs of the chest and forepaw.

Hence, viral vector delivery of mineral-targeted TNAP has potential to treat HPP with significantly reduced frequency of injections and cost of treatment.

## Concluding Remarks

 There has been substantial progress during the past decade in understanding TNAP and therefore in treating HPP patients. Clinical trials of EzRT using asfotase alfa (now approved as Strensiq™ in Japan, Canada, Europe, and the USA generally for pediatric-onset HPP) have shown skeletal, respiratory, and functional improvement as well as prevention of seizures in the most severe perinatal and infantile forms of the disease and attainment of good health in survivors of infantile HPP and with severe childhood HPP. Ongoing clinical trials are revealing aspects of HPP that we do not yet fully understand, such as its treatable muscle weakness, or that seemingly cannot be prevented such as craniosynostosis. Now that life-threatening and debilitating pediatric HPP is treatable using asfotase alfa, and the risks and benefits of this EzRT in managing HPP in adults require study. These patients may present unique challenges because vascular calcification can be a comorbidity of aging, diabetes mellitus, or chronic kidney disease. Binding of mineral-targeted TNAP to such sites of ectopic calcification could theoretically lead to cardiovascular complications [137]. It is not known if non-targeted ALPs will be useful alternatives. Safe viral vectors for delivery of mineral-targeted or soluble ALPs may streamline HPP treatment in the future.
